# A Nanocomposite Paste Electrode Sensor for Simultaneous Detection of Uric Acid and Bisphenol A Using Zinc Hydroxide Nitrate-Sodium Dodecylsulfate Bispyribac

**DOI:** 10.3390/s23208366

**Published:** 2023-10-10

**Authors:** Yulkifli Yulkifli, Widya Putri Yandes, Illyas Md Isa, Norhayati Hashim, Alizar Ulianas, Sharifah Norain Mohd Sharif, Mohamad Idris Saidin, Mohamad Syahrizal Ahmad, Siti Nur Akmar Mohd Yazid, Suyanta Suyanta, Ratno Nuryadi, Nurashikin Abd Azis

**Affiliations:** 1 Department of Physics, Faculty of Mathematics and Natural Sciences, Universitas Negeri Padang, Padang 25131, Indonesia; 2 Department of Chemistry, Faculty of Science and Mathematics, Universiti Pendidikan Sultan Idris, Tanjong Malim 35900, Perak, Malaysia; widyputri11@gmail.com (W.P.Y.); norhayati.hashim@fsmt.upsi.edu.my (N.H.); norain.sharif@fsmt.upsi.edu.my (S.N.M.S.); idris.saidin@fsmt.upsi.edu.my (M.I.S.); syahrizal@fsmt.upsi.edu.my (M.S.A.); snakmar@fsmt.upsi.edu.my (S.N.A.M.Y.); 3 Department of Chemistry, Faculty of Mathematics and Natural Sciences, Universitas Negeri Padang, Padang 25131, Indonesia; alizarulianas@fmipa.unp.ac.id; 4 Department of Chemistry Education, Faculty of Mathematics and Natural Science, Yogyakarta State University, Yogyakarta 55281, Indonesia; suyanta@uny.ac.id; 5 Center for Materials Technology, Agency for the Assessment and Application of Technology, Puspiptek Building #224, South Tangerang, Banten 15314, Indonesia; ratno.nuryadi@brin.go.id; 6 Department of Academic Affairs, North Borneo University College, Wisma Angkatan Hebat, 1 Borneo, Jalan Sulaman, Kota Kinabalu 88400, Sabah, Malaysia; nurashikin@nbuc.edu.my

**Keywords:** layered double hydroxide, modified paste electrode, uric acid, bisphenol A

## Abstract

The fabrication of a zinc hydroxide nitrate-sodium dodecylsulfate bispyribac modified with multi-walled carbon nanotube (ZHN-SDS-BP/MWCNT) paste electrode for uric acid and bisphenol A detection was presented in this study. Electrochemical impedance spectroscopy, chronocoulometry, square-wave voltammetry, and cyclic voltammetry were all used to examine the electrocatalytic activities of modified paste electrodes. The modified electrode’s sensitivity and selectivity have been considered in terms of the composition of the modifier in percentages, the types of supporting electrolytes used, the pH of the electrolyte, and square-wave voltammetry parameters like frequency, pulse size, and step increment. Square-wave voltammetry is performed by applying a small amplitude square-wave voltage to a scanning potential from −0.3 V to +1.0 V, demonstrating a quick response time and high sensitivity. The ZHN-SDS-BP/MWCNT sensor demonstrated a linear range for uric acid and bisphenol A from 5.0 µM to 0.7 mM, with a limit of detection of 0.4 µM and 0.8 µM, respectively, with good reproducibility, repeatability, and stability as well. The modified paste electrode was successfully used in the determination of uric acid and bisphenol A in samples of human urine and lake water.

## 1. Introduction

Uric acid (UA) is an end product of purine metabolism and is considered a species of great importance in human diagnosis. UA is important for the detection of abnormal levels of UA in the human body. Extremely high UA levels can build up in the body, and excessive amounts in bodily fluids can cause solid-state urate, which can cause kidney or gout stones, as well as hyperuricemia, which is a separate risk factor for the onset of cardiovascular and renal disease as well as Lesh–Nyhan syndrome. Meanwhile, multiple sclerosis, Parkinson’s disease, Alzheimer’s disease, and optic neuritis have all been associated with reduced UA concentration. Gout develops when sodium urate crystals accumulate in the tendons, bursae, soft tissues, and joints [1,2]. Urate, or UA’s salt, is the most common form of UA. UA crystal formation increases along with blood urate concentration. Women’s normal reference ranges for UA in blood are 1.5–6.0 mg/dL, while men’s are 2.5–7.0 mg/dL. Most daily UA disposal occurs via the kidneys. Therefore, it is important to monitor the concentration of UA in biological fluids so that it can be used as an early warning of the presence of UA in urine [3].

Bisphenol A (BPA) is most commonly used in the production of polycarbonate and epoxy resin in the chemical industry worldwide. BPA, with the chemical name 2,2-(4,4-dihydroxydiphenyl) propane, is an organic compound consisting of two phenolic rings connected by a single carbon carrying two methyl groups. Exposure to BPA triggers an adverse effect in the natural environment and brings major concern, as it induces negative effects on human health. BPA has been found to leach out into the environment from bottles, packaging, plastic plants, and landfill leachates when exposed to certain conditions, such as high temperatures (heating) or when cleansed with rough or acidic detergents. Consequently, BPA migrates into food and drinking water from a wide variety of food contact materials, and when humans routinely ingest trace amounts of BPA, it will accumulate in the body system [4].

Several analytical methods are applicable for the determination of BPA and UA, such as high-performance liquid chromatography, Fourier transform infrared spectroscopy, solid-phase extraction, chemiluminescence, colorimetric, and quartz crystal microbalance (QCM). There are several methods of determining UA or BPA using electrochemical methods, such as voltammetry, chromatography, and quartz crystal microbalance (QCM) sensor chips [5].

To the best of our knowledge, no other work has been reported on the simultaneous determination of UA and BPA using various analytical or electroanalytical methods. Some electroanalytical methods were reported for the simultaneous determination of UA and dopamine [6], UA, ascorbic acid, and dopamine [7,8], while the simultaneous determination of BPA and another analyte via electroanalytical methods was reported by some researchers [9,10,11].

The modification of electrodes offers remarkable advantages to electrochemical sensors as it resolves the slow kinetics of many plain or bare electrodes. Yet, the addition of layered double hydroxides (LDH) shows better performance in electrocatalysis and provides better detection of analytes by overcoming the poor response of analytes. LDH can obviously be an attractive material for modified electrodes in electrochemical analysis [12,13,14].

In this work, the doping of zinc hydroxide nitrate-sodium dodecylsulfate bispyribac, ZHN-SDS-BP (LDH) in multi-walled carbon nanotubes was developed for the simultaneous determination of UA and BPA. This study was carried out to determine the contents of UA and BPA in the release of wastewater reservoirs, direct urination into drains, and the leaching of BPA from plastic materials. The advantages of this sensor are high sensitivity, low cost, simple operation, and rapid analysis.

## 2. Materials and Methods

### 2.1. Chemicals and Reagents

All chemicals and reagents in this study were of analytical grade and used without further purification. Zinc hydroxide nitrate-sodium dodecylsulfate bispyribac (ZHN-SDS-BP) nanocomposite was synthesized according to the method previously reported [15]. The pH of phosphate-buffer solutions (PBS) was prepared by mixing the stock solution of 0.1 M potassium dihydrogen phosphate (KH_2_PO_4_) and 0.1 M dipotassium hydrogen phosphate (K_2_HPO_4_) (Merck, Germany). All reagents were of analytical grade and used as received without any further purification unless otherwise indicated.

### 2.2. Instruments

The deionized water from EASY pure LF, Barnstead, was used to prepare all the solutions. The characterization of ZHN-SDS-BP was carried out with a scanning electron microscope (SEM) and a transmission electron microscope (TEM) using a field emission scanning electron microscopy (FESEM) model Hitachi SU 8020 UHR (Tokyo, Japan). The pH measurements were conducted with a pH meter, an Orion 720A (MI, USA), equipped with a glass electrode. Cyclic voltammetry (CV) and square-wave voltammetry (SWV) were performed with the Potentiostat Gamry Series-G750 (Warminster, PA, USA), and the electrochemical data were obtained using three-electrode systems consisting of an Ag/AgCl electrode model MF-2052 from Bioanalytical System (West Lafayette, IN, USA) with a fiber junction as the reference electrode, a platinum wire as a counter/auxiliary electrode, and a ZHN-SDS-BP-modified multi-walled carbon nanotube (MWCNT) paste electrode as a working electrode. The electrochemical impedance spectroscopy (EIS) measurements were carried out on a Potentiostat/Galvanostat Gamry model Ref 3000 (PA, USA). Convincingly, the real sample validation was conducted by using high-performance liquid chromatography (HPLC, column, C-18; detector, UV-Vis (DAD) 280 nm) with the Agilent 1200 Infinity Series (Waldbronn, Germany).

### 2.3. Preparation of ZHN-SDS-BP Nanocomposite

The ZHN-SDS-BP nanocomposite was synthesized following the method of Sharif et al. [16]. We continuously stirred a mixture of 40 mL of 10 mmol SDS, 40 mL of 20 mmol Zn(NO_3_)_2_·6H_2_O, and 10 drops of 1M NaOH at room temperature in a nitrogen atmosphere. The pH was adjusted to a value of 6.5 by adding a few drops of 1 M HCI. The white slurry suspension was aged in an oil bath shaker at 70 °C for 24 h and then centrifuged. The precipitate was collected, and the ZHN-SDS was then dried in an oven at 60 °C. A BP was intercalated into the ZHN-SDS by dispersing 0.3 g of ZHN-SDS into 50 mL of 1 mmol BP in ethanol and deionized water (10:90). The precipitate, ZHN-SDS-BP was thoroughly washed with deionized water and dried in an oven at 60 °C.

### 2.4. Preparation of the MWCNTs/ZHN-SDS-BP Electrode

The fabrication of the proposed electrode was conducted by mixing 10 mg of ZHN-SDS-BP with 90 mg of MWCNT (10%, *w*/*w*), and two drops of paraffin oil that act as a binder were added to the mixture and mixed until a homogenized paste was obtained. Also, different ratios of ZHN-SDS-BP, such as 0%, 5%, and 15%, were prepared. The homogenized paste was then packed into the Teflon tubes (diameter = 2.0 mm, long 3.0 cm) and pressed with a small rod of stainless steel inside a micropipette tip until firmly fully packed. At one end of the tubing, mercury was placed, and copper wire was inserted in order to make electrical contact with the potentiostat.

### 2.5. Measurements of UA and BPA

The voltammetric measurements of the modified and unmodified paste electrode were performed in 0.1 M PBS (pH 6.0) at the desired concentration of UA and BPA. The voltammograms were recorded via a potential scan rate between −0.3 V to +1.0 V by utilizing SWV with a frequency of 180 Hz, a pulse height of 70 mV, and a step increment of 6 mV. All experiments were conducted at a room temperature of 25 ± 2 °C and in an air atmosphere.

### 2.6. Preparation of Real Sample Analysis

Urine samples of healthy volunteers were collected and stored immediately after collection in a refrigerator (±4 °C). Ten milliliters of the sample were centrifuged for 10 min at 1500 rpm. The residue was filtered out using filter paper (Whatman no. 42 with a diameter of pores 2.5 µm). Then, the volume of the solution was transferred into a 50.00 mL volumetric flask and diluted three times at a 1:3 ratio with 0.1 M PBS at pH 6.2. The diluted urine samples were spiked with different amounts of BPA and UA. Meanwhile, the lake waters were collected, and 1.0 mL of each sample solution was mixed with 9.0 mL of PBS buffer (pH 6.2) and then analyzed. The blank was prepared using a similar procedure without the addition of analytes.

## 3. Results and Discussion

### 3.1. Morphological Studies and Electrochemical Characterization

**Figure 1:**
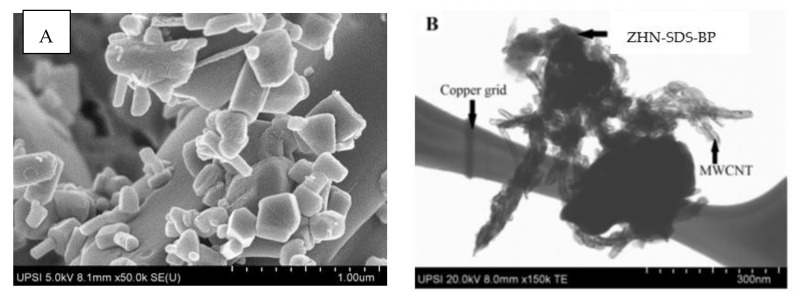
(**A**) SEM image of ZHN-SDS-BP and (**B**) TEM image of ZHN-SDS-BP/MWCNT.

The surface morphology of ZHN-SDS-BP and ZHN-SDS-BP on the wall of MWCNTs was observed using SEM and TEM, and the images are presented in Figure 1. ZHN-SDS-BP appears with an irregular and small particle size under SEM, as shown in Figure 1A. Meanwhile, the TEM analysis of the hybrids in Figure 1B shows that MWCNT is well dispersed in the presence of ZHN-SDS-BP. The results from SEM and TEM clearly reveal the existence of ZHN-SDS-BP and MWCNT.

**Figure 2:**
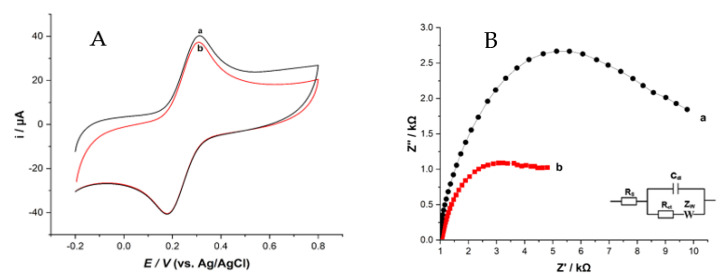
Cyclic voltammetry (**A**) and Nyquist plot (**B**) of the unmodified MWCNT and ZHN−SDS/MWCNT paste electrodes in 4.0 mM K_3_[Fe(CN)_6_]. Inset: Randle’s equivalent electrical circuit system. (a) Unmodified MWCNT and (b) ZHN−SDS−BP/MWCNT.

The CV and EIS were carried out to study the electrochemical characteristics of the modified electrodes by using K_3_Fe(CN)_6_ solutions. A well-defined cyclic voltammogram, displayed in Figure 2A, shows the CV response of 4.0 mM K_3_Fe(CN)_6_ containing 0.1 M KCl as a supporting electrolyte from −0.3 V to +1.0 V at a scan rate of 100 mV s^−1^. The bare MWCNT exhibited an anodic peak current equal to 18.98 µA and a cathodic peak current equal to −22.59 µA. After modifying, the redox peak current for the ZHN-SDS-BP/MWCNT paste electrode showed a slight increase in the anodic peak current equal to 21.76 µA and the cathodic peak current equal to −24.81 µA. The increases in the anodic and cathodic peak currents are due to the bispyribac (BP) anions exchanging capacities in the modifier. In addition, the peak-to-peak (ΔEp) separation of the ZHN-SDS-BP/MWCNT paste electrode also decreased from 320.1 mV to 300.1 mV. This suggests that the relatively fast electron transfer at the modified electrode, due to its excellent conductivity and increased electron transfer rate at the electrode surface, might improve the sensitivity of the ZHN-SDS-BP-modified MWCNT paste electrode.

The EIS spectrum was employed to evaluate the electronic properties of the ZHN-SDS-BP-modified MWCNT paste electrode. Figure 2B presents the Nyquist diagrams of (a) bare MWCNT and (b) the ZHN-SDS-BP/MWCNT paste electrode in 4.0 mM K_3_[Fe(CN)_6_] containing 0.1 M KCl at frequencies ranging from 10 MHz to 1 Hz. The spectrum consists of a semicircle portion and a linear part. The diameter of the semicircle in the Nyquist diagram indicates the electron transfer resistance (R_ct_), which works as a kinetic control parameter for electron transfer at the electrode interface, and the linear component depicts the diffusion process [17].

The semicircle diameter, which stands for electron transfer resistance (R_ct_), is a kinetic control parameter for interfacial electron transfer at the electrode. At the same time, the linear portion symbolizes the diffusion process. A low R_ct_ (0.2610 kΩ) using modified ZHN-SDS-BP/MWCNT and high R_ct_ (8.50 kΩ) using bare MWCNT suggested that the modified ZHN-SDS-BP/MWCNT electrode has a higher conductivity and a faster electron transfer process than bare MWCNT. From the R_ct_ data obtained, the electron transfer across the interface of the ZHN-SDS-BP/MWCNT and the bare MWCNT paste electrode could be evaluated through the apparent electron transfer rate constant (k_app_) determined from Equation (1):

k_app_ = RT/F^2^R_ct_ CA (1)

where R is the gas constant (8.314 J mol^−1^ K^−1^), T is the absolute temperature of the system (298 K), F is the Faraday constant (96,485 Cmol^−1^), A is the estimated area of the electrode, and C is the concentration of the K_3_[Fe(CN)]_6_ solution.

For bare MWCNT, the k_app_ value was 2.12 × 10^−4^ cms^−1^. The electrode was modified by adding ZHN-SDS-BP and MWCNT, increasing the k_app_ value to 5.33 × 10^−3^ cms^−1^. Large k_app_ values showed a lower R_ct_, which was evidence of a quicker electron transfer process between the electrode surface of the modified ZHN-SDS-BP electrode and the K_3_[Fe(CN)_6_] solution.

### 3.2. Electrochemical Responses of UA and BPA

**Figure 3:**
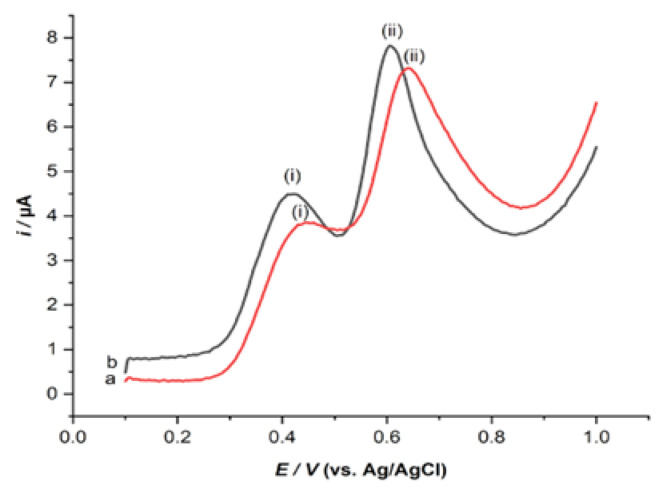
SW voltammogram of 0.1 mM (i) UA and (ii) BPA with the (a) unmodified MWCNT and (b) ZHN−SDS−BP/MWCNT paste electrodes.

The SWV responses of 0.1 mM UA and BPA in the presence of 0.1 M PBS at pH 6.0 were registered on the ZHN-SDS-BP/MWCNT paste electrode and the unmodified MWCNT paste electrodes, as shown in Figure 3. It can be observed that the oxidation peak current of UA and BPA on the ZHN-SDS-BPMWCNT paste electrode was 1.50 µA and 3.76 µA, respectively. The peak currents of UA and BPA were higher than those of 0.99 µA and 3.00 µA at a pulse size = 60 Mv, step size = 5 mV, and frequency = 200 Hz. The peak current was increased by 52% and 25% for UA and BPA, respectively. This demonstrated that the electrochemical response was seen for ZHN-SDS-BP/MWCNT and that electrochemically active anionic impurities affected the rise in the oxidation peak current [18]. It has been demonstrated that the suggested sensor had a synergistic impact that significantly increased electron transfer kinetics at the electrode.

### 3.3. Optimization of Variables of ZHN-SDS-BP/MWCNT Paste Electrode

#### 3.3.1. The Effect of ZHN-SDS-BP Composition Percentage

**Figure 4:**
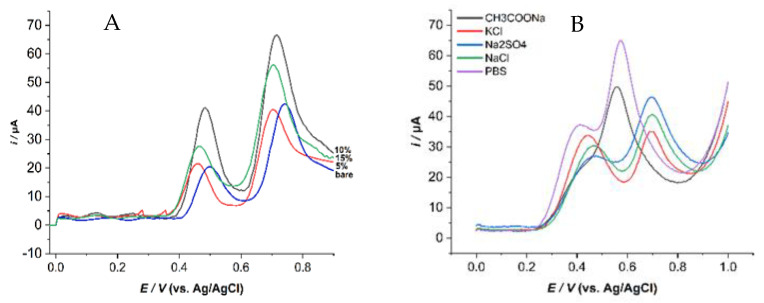
The SWV of (**A**) different composition ratios and (**B**) types of supporting electrolytes on the peak currents of 0.1 mM UA and BPA in the presence of 0.1 M BPS at pH 6.2 (pulse size = 60 mV; step size = 5 mV; frequency (*f*) = 200 Hz).

It is well known that the electrochemical characteristics of the modified electrode depend greatly on the paste composition. The effect of the composition ratios of ZHN-SDS-BP, used as a modifier in carbon paste composition, on the electrochemical response of 0.1 mM of UA and BPA solutions was investigated using SWV. Figure 4A shows the electrodes with different percentages of modifiers (0%, 5%, 10%, and 15%) (*w*/*w*), which were prepared and examined for their voltammetric signals under identical conditions.

The results show that when the amount of modifier was increased from 0 to 15%, the peak current intensity was increased until it reached the maximum value obtained at 10% of the ZHN-SDS-BP paste electrode. In higher compositions of the modifier, the current significantly decreased, and the electrode became less conductive and unstable. These conditions can be presumably due to the reduction in the conductive area, which is a carbon particle at the surface electrode [19].

Furthermore, the peak current started to decrease after 15% of the ZHN-SDS-BP, probably due to the repelling effect of the hydrophobicity of the pasting liquid (paraffin oil) that blocks the electron penetration of analytes towards the electrode surface. Yet, the amount of paraffin oil used has a substantial impact on the conductivity. Thus, the 10% composition of the ZHN-SDS-BP modifier was selected as the optimum composition with the highest peak current and was used for further research and study [20,21].

#### 3.3.2. The Effect of Supporting Electrolyte Types

The rationale for using supporting electrolytes is to inhibit the migration of electro-active species via electrostatic attractions to the electrodes and thereby obtain diffusion-controlled currents [22]. Thus, the supporting electrolytes chosen for this experiment vary from sodium chloride (NaCl) to potassium chloride (KCl), sodium acetate (CH_3_COONa), sodium sulfate (Na_2_SO_4_), and PBS. Figure 4B shows that among these five supporting electrolytes, PBS showed the highest peak current response of SWV; hence, 0.1 M PBS was chosen as a supporting electrolyte for the subsequent studies.

#### 3.3.3. The Effect of pH on Supporting Electrolytes

**Figure 5:**
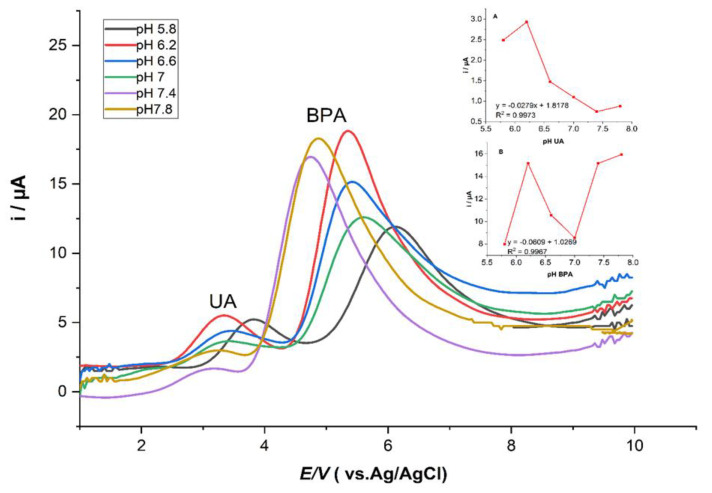
SWV curves of UA and BPA and peak potential vs. pH (5.8 to 7.8) of 0.1 mM (**A**) UA and (**B**) BPA in 0.1 M PBS. Inset: Linear plot of peak current vs. pH of UA and BPA.

The effect of the pH values on the current responses in modified ZHN-SDS-BP/MWCNT paste electrodes was investigated for 0.1 mM UA and BPA in 0.1 M PBS over a range from 5.8 to 7.8 (Figure 5). The results show that the oxidation peak currents of UA and BPA were optimal at pH 6.2. A great decrease in the oxidation peak current was observed after exceeding pH 6.2, suggesting that the oxidation reaction of UA and BPA was kinetically more favorable in an acidic medium [23,24]. Then, a pH of 6.2 was selected for the subsequent analytical study.

**Figure 6:**
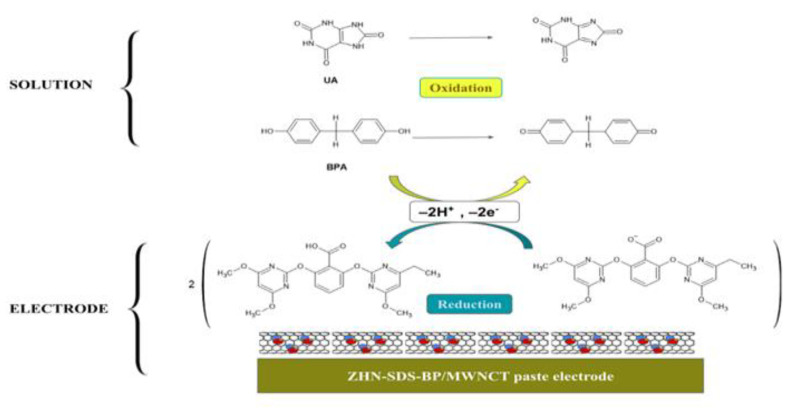
The illustration of the entire possible reaction and the mechanism of the UA and BPA reaction on the surface of the ZHN−SDS−BP/MWCNT paste electrode.

The selectivity of the paste electrode ZHN-SDS-BP/MWCNT was based on the configuration and design of the modifier used. Meanwhile, BPA is an electro-active material due to containing phenolic hydroxyl groups. The potential reaction mechanism of the ZHN-SDS-BP/MWCNT paste electrode and BPA solution is shown in Figure 6. In the solution, BPA was oxidized by releasing two electrons and protons. Meanwhile, the ZHN-SDS-BP/MWCNT interlayer accepted the electrons and protons on the electrode surface.

#### 3.3.4. The Effect of Square-Wave Voltammetry Parameters

Since they control the signal’s intensity and the method’s sensitivity, the SWV parameters are highly significant in SWV. To determine the optimal resolution and sensitivity of analytes towards electrodes, the SWV parameters have been evaluated. To achieve the greatest sensitivity at a rapid scan rate, the effects of factors, including frequency, pulse size, and step size, were examined in a solution containing 1.0 mM UA and BPA in the presence of 0.1 M PBS at pH 6.2. Figure S1, Figure S2, and Figure S3 show the SW voltammogram plots of frequency, pulse size, and step size, respectively.

The influence of frequency was analyzed by raising the frequency from 40 Hz to 240 Hz, as shown in Figure S1. The peak current increased up to 200 Hz and started to decrease at frequencies over 200 Hz. Aside from the frequency, the effect of the square-wave pulse size was also investigated in the range of 10 mV to 60 mV (Figure S2). The result revealed that the highest peak current was obtained at a pulse size of 60 mV. The influence of step size was evaluated from 1 mV to 5 mV. As shown in Figure S3, the step size of 5 mV displayed the highest peak current, and further increases in the step size caused the peak current to decrease. Overall, similar patterns were observed for frequency, pulse size, and step size. Therefore, the optimum parameters used for subsequent experiments were at a frequency of 200 Hz, a pulse size of 60 mV, and a step size of 5 mV.

### 3.4. Chronocoulometric Studies

The electrochemically effective surface area (A) of unmodified MWCNT and ZHN-SDS-BP-modified MWCNT paste electrodes was investigated via chronocoulometry. The measurement of charge (coulombs) as a function of time (second), noted as Q (t), was calculated according to Equation (2), given by Anson [25].

**Figure 7:**
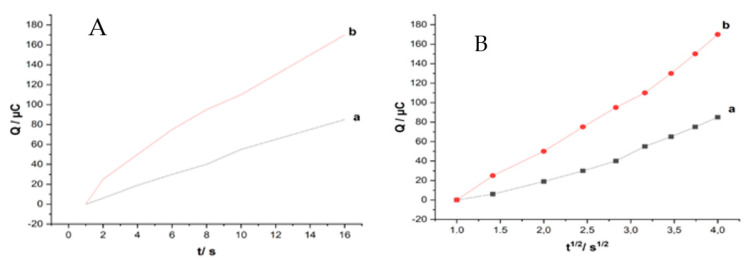
Graph of Q vs. t (**A**) and Q vs. t^1/2^ (**B**) at (a) unmodified MWCNT and (b) ZHN−SDS−BP/MWCNT paste electrodes in 4.0 mM K_3_[Fe(CN)_6_].

Q (t) = 2nFAc + Q_dl_ + Q_ads_ (2)

where Q (t) is the charge, n is the number of electrons transferred, F is the Faraday constant (96485 Coulombs/mole), c is the concentration of the substrate, D is the diffusion coefficient of K_3_[Fe(CN)_6_] (7.6 × 10^−6^ cm^2^ s^−1^ at 25 °C), and t is the time (s). Q_dl_ is the double-layer charge, which could be eliminated by background subtraction, and Q_ads_ is the Faradaic charge. According to the plots of Q vs. t^1/2^ (Figure 7A), the Q value of unmodified MWCNT was 49.81 × 10^−6^ t^1/2^ − 45.14 × 10^−6^ and 71.17 × 10^−6^ t^1/2^ − 62.09 × 10^−6^ for ZHN-SDS-BP.

The effective surface area (A) of unmodified MWCNT and ZHN-SDS-BP paste electrodes was determined via double-potential-step chronocoulumetry using 4.0 mM K_3_[Fe(CN)_6_] in the presence of 0.1M KCl as a model complex. From the slopes of Q vs. t^1/2^ (Figure 7B), the effective electrochemical surface area for the unmodified MWCNT and ZHN-SDS-BP paste electrodes was calculated as 0.046 cm^2^ and 0.059 cm^2^, respectively, indicating the A of the ZHN-SDS-BP/MWCNT paste electrode increased obviously after the addition of the ZHN-SDS-BP, leading to improve the currents’ response towards K_3_[Fe(CN)_6_]. The results showed that the modification of the MWCNT paste electrode with ZHN-SDS-BP improved the effective surface area of the electrode.

**Figure 8:**
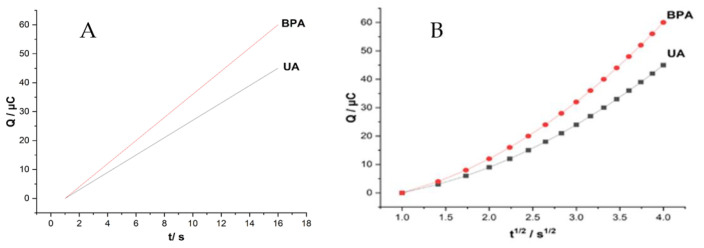
Graph of (**A**) Q vs. t and (**B**) Q vs. t^1/2^ with ZHN−SDS−BP/MWCNT paste electrodes in 0.1 mM UA and BPA.

The chronocoulometric experiments were also carried out on modified ZHN-SDS-BP/MWCNT in 0.1 M PBS at pH 6.2 in the presence and absence of 0.1 mM UA and BPA, respectively. A plot of charge (Q) against the square root of time (t^1/2^) (Figure 8A) was obtained after background subtraction and exhibited a slightly linear relationship with UA and BPA. From the Anson equation, the Q values for UA and BPA were 19.14 × 10^−6^ t^1/2^ − 44.73 × 10^−6^ and 18.62 × 10^−6^ t^1/2^ − 39.48 × 10^−6^, respectively.

As a result, 1.44 × 10^−1^ cm^2^ s^−1^ and 1.48 × 10^−1^ cm^2^ s^−1^, respectively, were determined as the diffusion coefficients, D, for UA and BPA. The adsorption capacities, Γs for UA and BPA, were calculated using the Cottrell equation, Q_ads_ = nFAΓs, as 3.47 × 10^−9^ mol cm^2^ and 3.92 × 10^−9^ mol cm^2^, respectively (Figure 8B). According to these values, the ZHN-SDS-BP/MWCNT paste electrode has a good capability for UA and BPA adsorption.

### 3.5. Calibration Data, Limit of Detection, Reproducibility, Repeatability, and Stability

**Figure 9:**
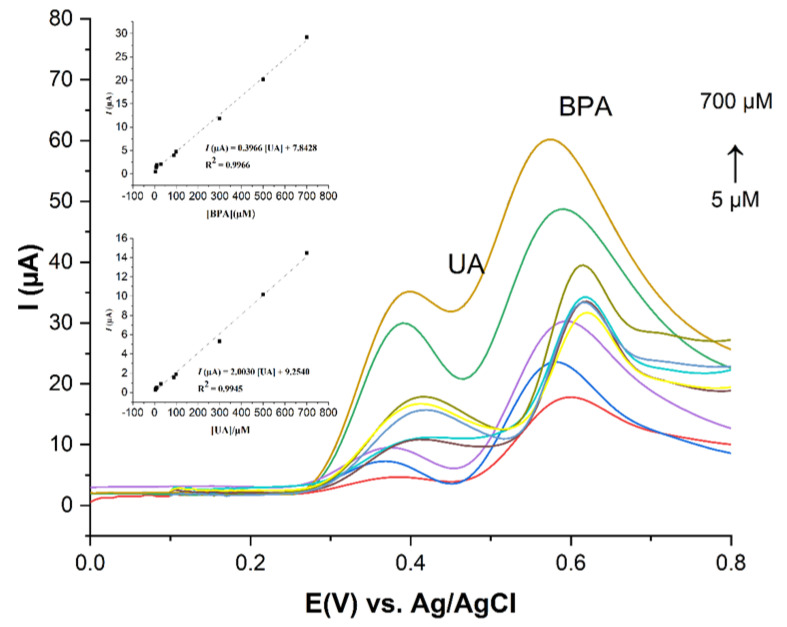
SWV curves of UA and BPA solution at various concentrations. Inset: Linear plot of peak current vs. concentration of UA and BPA.

A good linear relationship between the anodic peak current and the concentration of BPA and UA can be seen in Figure 9. As can be seen, the anodic peak current increased with the increasing concentrations of UA and BPA from 5 µM to 700 µM, respectively. The peak current was linearly proportional to the concentrations of UA and BPA, and this can be described by the linear regression equation, expressed as I = −2.0030 [UA] + 9.2540 (R^2^ = 0.9945) and I = 0.3966 [BPA] + 7.8428 (R^2^ = 0.9966), where I, [UA], and [BPA] represent the response current (µA), uric acid, and bisphenol A concentrations (µM), respectively. The limits of detection, calculated based on 3σ/S (σ is the standard deviation of the response, and S is the slope of the calibration curve), were 0.4 µM and 0.8 µM for UA and BPA, respectively. The high sensitivity and limit of detection of the ZHN-SDS BP/MWCNT paste electrode to UA and BPA are comparable to those obtained for several other electrodes by different electroanalytical methods, and this is presented in Table 1. It indicated that the ZHN-SDS-BP/MWCNT paste electrode could provide a good platform for the effective analytical detection of UA and BPA.

The reproducibility, repeatability, and storage stability of the sensor for determination of 1.0 × 10^−4^ M UA and BPA were investigated by using SWV under optimal experimental conditions. The reproducibility of the sensor was examined using three electrodes that were prepared the same way as the previous electrode to detect UA and BPA in the 0.1M PBS solution. The reproducibility of the method was demonstrated by the relative standard deviation (RSD), where the RSD is estimated by dividing the standard deviation by the average and expressed in the form of a percentage. The RSDs for the UA and BPA obtained from the sensors were 0.87% and 2.59%, respectively, which indicates that all the sensors possess similar current responses and are stable.

The repeatability was evaluated using five replicate measurements (*n* = 3) with the same electrode. The electrode was prepared with the same procedure, and the RSDs obtained for the five replicate measurements of UA and BPA were 3.01% and 3.34%, respectively, indicating that they can be used for repeated measurements [28].

The storage stability of the electrodes was investigated by measuring the current responses of UA and BPA. The electrode was stored in the laboratory at room temperature (25 °C) for 4 weeks. The current response changes were measured during the first week. Figure S4 shows the response current values retained from the original responses for both UA and BPA. After four weeks, the electrode demonstrated excellent stability, maintaining 93.9% and 95.8% of the initial responses for UA and BPA, respectively, indicating that good long-term stability could be obtained from the electrode for the detection of UA and BPA [13].

### 3.6. Interferences Studies

**Figure 10:**
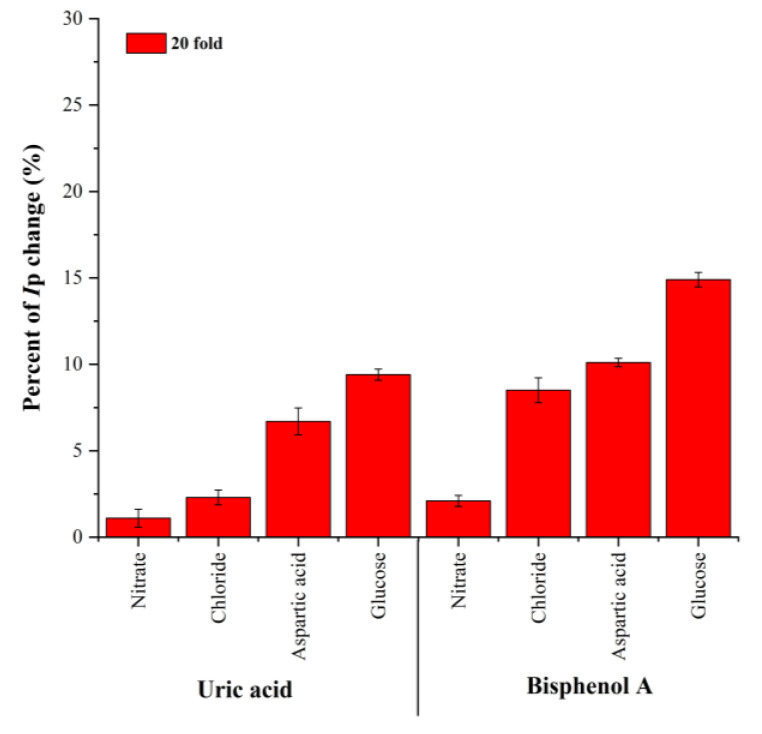
The response of 20-fold concentrations of possible interferences on the determination of 0.1 mM UA and BPA in the ZHN-SDS-BP/MWCNT paste electrode.

The selectivity of the electrode was studied by evaluating the effect of interferences in the detection of UA and BPA. Under optimum conditions, several possible interferences, such as nitrate, chloride, aspartic acid, and glucose, were added at a 20-fold higher concentration into 1 × 10^−4^ M UA and BPA in 1 × 10^−1^ M PBS at pH 6.2. The result indicates that the presence of selected interferences at a 20-fold excess did not interfere, with the exception of glucose (Figure 10). This was presumably due to competition between the hydroxyl groups from the glucose and the UA and BPA.

As shown in Figure 10, the relative signal changes of most of the interferences were less than 15%. The smaller percentage of relative signal changes indicated that the current response was not much affected by the existence of interferences at 20-fold. The ZHN-SDS-BP/MWCNT paste electrode showed a good anti-interference ability and was able to be employed in the determination of UA and BPA.

### 3.7. Real Sample Analysis

The modified ZHN-SDS-BP/MWCNT paste electrode was applied to evaluate the analytical applicability in real sample analysis. Employing the conventional standard addition method to measure the concentrations of UA and BPA in a urine sample made it possible to examine the validity of the data obtained using the ZHN-SDS-BP/MWCNT paste electrode. To evaluate electrode recoveries, known UA concentrations were spiked into the urine sample. This was based on the repeated SWV response performing triplicate (*n* = 3) measurements for the diluted samples that were spiked with specified concentrations of UA and BPA. The results of the percent recoveries were found effective for real sample analysis (Table 2). The ZHN-SDS-BP/MWCNT paste electrode shows remarkable recovery (89.1 to 98.3) for the determination of UA and BPA in urine and lake water samples, indicating a high degree of accuracy of the proposed method. Table 3 shows the results obtained by the proposed sensor and those of HPLC.

**Figure 11:**
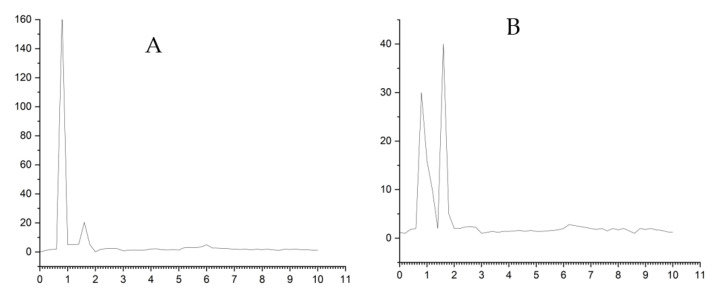
Graph of (**A**) HPLC chromatogram of urine sample and (**B**) HPLC chromatogram of lake sample. (Mobile phase, acetonitrile/water (55:45); injection volume, 10 µL; flow rate, 1.5 mL/min; column, C-18; detector, UV-Vis (DAD) 280 nm).

For comparison purposes, measurements of the urine and lake water samples were also obtained using HPLC (Figure 11). In order to investigate the correlation between the outcomes of the SWV and HPLC procedures, an independent *t*-test was conducted at a 95% level of confidence. Since *p*-values (0.10) and (0.17) for the urine and lake water samples are greater than the significance level *p* > 0.05, the null hypothesis failed to be rejected, indicating there is no significant difference between the data obtained from the SWV and HPLC techniques, as shown in Table 3. The quantity of UA and BPA in the urine and lake water that was found using the proposed ZHN-SDS-BP/MWCNT paste electrode was in good agreement with that found via HPLC, as seen in Figure 11. Therefore, the proposed method has great potential for use as a reliable method for monitoring UA and BPA in urine and lake water.

## 4. Conclusions

In this experiment, the sensing material was simple, highly sensitive, and reasonably priced and was proposed for the testing of UA and BPA with a low LOD. These useful sensing electrode characteristics were developed through the combination of the unique properties of ZHN-SDS BP and MWCNT. Based on electrochemical studies, the effective electrochemical surface area, and the high adsorption capacity of UA and BPA, the ZHN-SDS-BP/MWCNT electrode paste exhibits significant catalytic activity for UA and BPA with higher peak currents.

With good selectivity, stability, and repeatability results, the produced electrodes demonstrated considerable electrocatalytic activity against UA and BPA oxidation, demonstrating that the ZHN-SDS BP/MWCNT paste electrode is a promising candidate for real-world uses. In lakes and urine samples, the ZHN-SDS-BP/MWCNT paste electrode demonstrated excellent recovery for UA and BPA.

## Figures and Tables

**Table 1:** Comparison between the current work and some reported sensors for the simultaneous determination of UA and BPA with other analytes.

Analyte	Modifier/Electrode	LWR (µM)	LOD (µM)	Ref.
UA + dopamine	Fe_3_O_4_/GO	1–100; UA	0.410	[7]
UA + dopamine + ascorbic acid	Pt NSs/C60/GCE	9.5–1187; UA	0.630	[26]
UA + dopamine + ascorbic acid	Pt/RGO	10–130; UA	0.450	[27]
BPA + paracetamol + dopamine	IL/GCE	2–100; BPA	0.028	[12]
BPA + H_2_O_2_	Polyterthiophene aerogel/SPE	1–200; BPA	-	[13]
BPA + H_2_O_2_	Palladium/Polyethlyenimine aerogel/SPE	1–500; BPA	0.025	[15]
BPA + UA	ZHN-SDS-BP/MWCNT	5.0–700; UA, BPA	0.371, 0.815	This work

LWR: linear working range; LOD: limit of detection; SPE: screen-printed electrodes; GO: graphene oxide; RGO: reduced graphene oxide; GCE: glassy carbon electrode; IL: ionic liquid.

**Table 2:** Recovery of UA and BPA in urine and lake water (*n* = 3).

Sample	Detected (μM)		Added (μM)		Found (μM)		Recovery (%)	
	UA	BPA	UA	BPA	UA	BPA	UA	BPA
Urine 1	25.54	N.D	4.0	12.0	28.13	11.86	95.23	98.30
Urine 2	24.10	N.D	4.0	12.0	26.64	10.97	94.80	91.41
Lake water 1	N.D	N.D	10.0	10.0	8.91	9.33	89.10	93.30
Lake water 2	N.D	N.D	10.0	10.0	9.56	9.61	95.60	96.10

N.D.: not detected.

**Table 3:** Validation of UA and BPA determination via the proposed method and HPLC (*n* = 3).

Samples	Method	Mean (µM)	Std. Deviation (µM)	Recovery (%)		Sig. (2-Tailed)
Urine (UA)	SWV	16.71	0.41	90.11	95.45	
	HPLC	15.04	0.35	89.31	89.60	0.10
Lake water (BPA)	SWV	20.01	0.57	83.67	91.10	
	HPLC	19.14	0.40	89.77	92.71	0.17

## Data Availability

The data presented in this study are available on request from the corresponding author.

## Supplementary Materials

The following supporting information can be downloaded at: https://www.mdpi.com/article/10.3390/s23208366/s1, Figure S1: The SWV of 0.1 mM UA and BPA at different frequency in the presence 0.1 M PBS at pH 6.2. Figure S2: The SWV of 0.1 mM UA and BPA at different pulse size in the presence of 0.1 M PBS at pH 6.2. Figure S3: The SWV of 0.1 mM UA and BPA at different step size in the presence of 0.1 M PBS at pH 6.2. Figure S4: Stability of the ZHN-SDS-BP/MWCNT paste electrode for determination of 0.1 mM UA (A) and BPA(B) in the presence of 0.1 M PBS at pH 6.2. Figure S5: Voltammograms of real samples in the presence of 0.1 M PBS at pH 6.2 (*n* = 3).
