# Supplementary material for: A Nanocomposite Paste Electrode Sensor for Simultaneous Detection of Uric Acid and Bisphenol A Using Zinc Hydroxide Nitrate-Sodium Dodecylsulfate Bispyribac
Source: Sensors (Basel). 2023 Oct 10;23(20):8366. doi: 10.3390/s23208366 (PMC10610553; doi:10.3390/s23208366)
Supplement: Supplementary file 1 [file sensors-23-08366-s001.zip › sensors-2541913-supplementary.pdf]

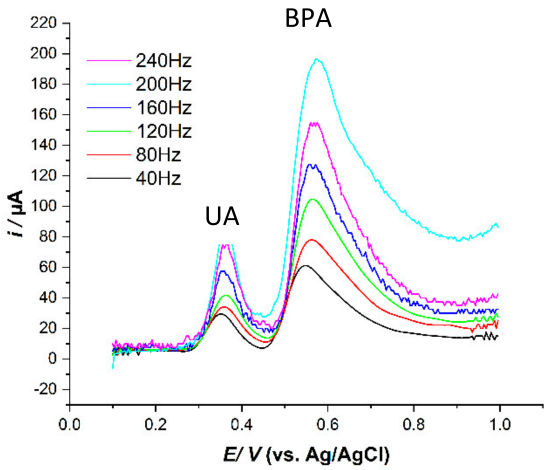

Figure S1. The SWV of 0.1 mM UA and BPA at different frequency in the presence 0.1 M PBS at pH 6.2.

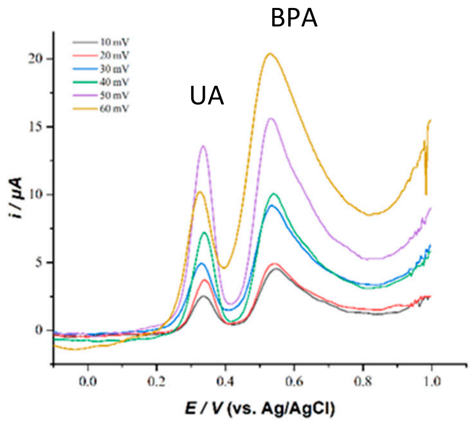

Figure S2. The SWV of 0.1 mM UA and BPA at different pulse size in the presence of 0.1 M PBS at pH 6.2.

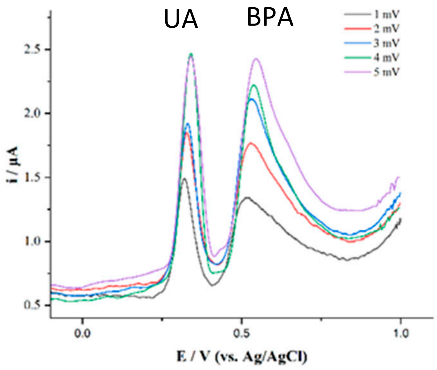

Figure S3. The SWV of 0.1 mM UA and BPA at different step size in the presence of 0.1 M PBS at pH 6.2.

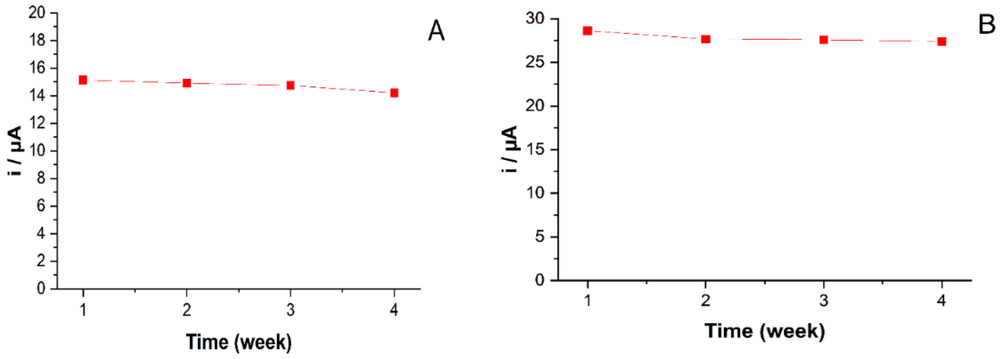

Figure S4. Stability of the ZHN-SDS-BP/MWCNT paste electrode for determination of 0.1 mM UA (A) and BPA(B) in the presence of 0.1 M PBS at pH 6.2.

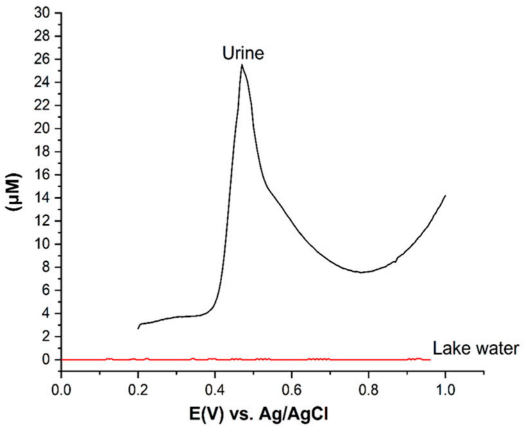

Figure S5. Voltammograms of real samples in the presence of 0.1 M PBS at pH 6.2 (n = 3).
